# A Treatise on Fevers

**Published:** 1861

**Authors:** 


A Treatise on Fevers.—Selections from a course of Lectures on Fever, being part of a course of Theory and Practice of Medicine delivered by Robert D. Lyons, K.C.C., M.B.T.C.D., L.K.Q.C.P.I., L.R.C.S.I., M.R.I.A., Physician to Jervis Street Hospital, etc., etc., and late Pathologist-in-Chief to the British army in the Crimea. pp. 362.

A scholarly, well written work, by a new writer, and demanding a more discriminating review than we have had time to qualify for by a careful perusal. Of Sir Robert Lyons, knighted, we understand, for services during the memorable Crimean campaigns, we do not remember to have before heard, although his multiple titles, impressing almost an entire alphabet into initial service, would seem to indicate his position at home as by no means insignificant. We have glanced over his introductory chapters with no small pleasure at their fluency, ornate, yet simple style, and dignified and learned tone. The sections on *Typhoid Fever*, *Yellow Fever* and *Typhoid Fever* as it occurred in the Crimea, will possess increased interest at the present juncture, as the views and observations of one who occupied the distinguished position of Pathologist-in-Chief to the British army, during campaigns, in which 10,000 of its flower were carried off by diseases in seven months—an immense proportion of which was the result of fever and the sequelæ of fever. The opportunities for investigating the pathology of this class of diseases seem to have been very extensively and intelligently improved; and a large number of illustrative cases are cited, exhibiting the most marked forms of typhoid fever, the several conditions of the enteric lesions and the various morbid processes associated with it in its advanced stages,—*materiel* for which was abundantly supplied in the large hospitals before Sebastopol and at Scutari.

Philadelphia: Blanchard & Lea. Chicago: Wm. B. Keen.

